# Antenatal care and breastfeeding practices in Sub-Saharan Africa: an analysis of demographic and health surveys

**DOI:** 10.1186/s12884-025-07188-w

**Published:** 2025-01-27

**Authors:** Bolanle Olapeju, Michael Bride, Mariam Wamala, Deborah Atobrah, Elizabeth H. Lee, Zoé M. Hendrickson

**Affiliations:** 1 Department of Preventive Medicine and Biostatistics, Uniformed Services University of the Health Sciences, Bethesda, MD USA; 2 Johns Hopkins Center for Communication Programs, Baltimore, MD USA; 3 National Malaria Control Program, Ministry of Health, Kampala, Uganda; 4 Centre for Gender Studies and Advocacy, Institute of African Studies, University of Ghana, Accra, Ghana; 5 Department of Pediatrics, Uniformed Services University of the Health Sciences, Bethesda, MD USA; 6 Department of Behavioral and Community Health Sciences, University of Pittsburgh School of Public Health, Pittsburgh, PA USA

**Keywords:** Antenatal care, Breastfeeding, Exclusive, Immediate, Sub-saharan Africa, WHO policy

## Abstract

**Background:**

The World Health Organization (WHO) recommends immediate breastfeeding (within the first hour after birth) and exclusive breastfeeding (for the first six months of life), particularly in low-resource settings such as sub-Saharan Africa. In 2016, WHO updated its antenatal care (ANC) guidelines, recommending at least eight (8+) ANC contacts during pregnancy to improve maternal and child health outcomes. This study investigates i) trends in breastfeeding practices across sub-Saharan Africa following the rollout of the revised WHO 2016 ANC policy and ii) the relationship between ANC uptake and exclusive or early breastfeeding.

**Methods:**

We performed a secondary analysis of Demographic and Health Survey data from 19 countries, from 2018-2023. Key variables included exclusive breastfeeding, early initiation of breastfeeding (within one hour of birth), and the number of ANC contacts (categorized into 0-3, 4-7, and 8 + visits) among mothers with a live birth in the six months preceding the survey.

**Results:**

Exclusive breastfeeding rates ranged from 19% in Gabon to 81% in Rwanda (median = 53%), while early initiation of breastfeeding ranged from 32% in Senegal to 85% in Rwanda (median = 60%). The percentage of women with 8 + ANC contacts ranged from 0.3% in Rwanda to 39% in Ghana (median = 4%). Women with 8 + ANC contacts did not show increased odds of early initiation (aOR: 0.94; 95% CI: 0.84, 1.05) or exclusive breastfeeding (aOR: 0.85; 95% CI: 0.76, 0.94) compared to women with 4-7 contacts.

**Discussion:**

These findings reveal low rates of 8 + ANC contacts against a backdrop of suboptimal breastfeeding practices across sub-Saharan Africa. Furthermore, the results suggest limited additional benefits of 8 + ANC contacts over 4-7 contacts in promoting immediate and exclusive breastfeeding, as recommended by the WHO 2016 ANC policy. Urgent efforts are needed to promote ANC uptake and improve the quality of ANC contacts through behavior change interventions and complementary health service delivery. Sub-national, national, and global stakeholders should prioritize these interventions.

## Background

### Introduction

Breastfeeding is widely recognized as one of the most cost-effective and impactful interventions for improving infant health and reducing child mortality, especially in low-resource settings such as sub-Saharan Africa [1]. The World Health Organization (WHO) and UNICEF recommend exclusive breastfeeding (EBF), meaning feeding an infant solely breast milk for the first six months of life [2] and early initiation or immediate breastfeeding (IBF), defined as breastfeeding within the first hour after birth with no other foods or liquids are provided, including water [3]. Furthermore, infants should be breastfed on demand – that is, as often as the child wants, day and night [4].

### Benefits of breastfeeding

The benefits of immediate and exclusive breastfeeding (IEBF) are extensive for both infants and mothers. Infants who are exclusively breastfed receive vital nutrients and immune protection, which help guard against common health issues in low-resource settings, like diarrhea [5] and respiratory infections [6]. Research shows that breastfed infants are less prone to illnesses, experience better cognitive development, and face a lower risk of obesity and diabetes later in life [7]. For mothers, IEBF facilitates postpartum recovery, reduces the likelihood of breast and ovarian cancers, and assists in natural birth spacing, which is especially advantageous in areas with limited healthcare access [7]. Moreover, IEBF strengthens the maternal bond with the infant, contributing to the emotional well-being of both the mother and child [8]. Economically, insufficient EBF rates lead to higher healthcare costs due to increased infant illnesses [9], while the financial burden of purchasing formula places additional pressure on families, particularly in resource-constrained settings [1].

### Trends in breastfeeding

Despite these well-documented benefits, global trends are below the World Health Assembly 2025 targets for immediate and exclusive breastfeeding—70% and 50% respectively [10]. Globally, only 46% of newborns are immediately breastfed, while 48% of infants under six months of age are exclusively breastfed [11]. In sub-Saharan Africa, these trends vary significantly across countries. Immediate breastfeeding rates range from 16% in Chad to 93% in Eritrea, while exclusive breastfeeding rates range from 5% in Gabon to 81% in Rwanda [10]. Several sub-Saharan African countries have made notable strides in IEBF. Cameroon, Côte d’Ivoire, Djibouti, Gabon, Guinea, Madagascar, Mali, Mozambique, Nigeria, Sierra Leone, and Somalia all saw at least a 10-percentage-point increase in exclusive breastfeeding rates between 2017 and 2023 [11].

The low rates of IEBF may be due to several factors. Mothers may have some misconceptions about breastfeeding [1]. Studies suggest that while 84% of surveyed mothers were aware of exclusive breastfeeding, only 34% agreed that immediate breastfeeding was important [12]. Qualitative reviews suggest that immediate breastfeeding is hindered by breastfeeding difficulties [13] and misconceptions, such as beliefs that mothers lack milk [14], babies need to sleep immediately after birth, or that babies must show signs of hunger before being fed [15]. Relatedly, exclusive breastfeeding was associated with older maternal age [16], increased maternal education [17] and behavior change interventions such as breastfeeding counselling [18], nutrition enhancement [19] and education programs [20, 21].

### Factors associated with breastfeeding

In sub-Saharan Africa, breastfeeding is also impacted by community level factors such as residence in rural compared to urban areas [22], prevailing cultural norms [23], lack of maternal support [24] or community assets [25] and societal pressures for women to return to work shortly after childbirth [26]. These challenges are further compounded by the absence of effective legal policies, such as insufficient parental leave and inadequate breastfeeding breaks at work [26]. Finally, health care factors particularly in delivery and pregnancy period can directly impact IEBF [27]. These include facility delivery [27, 28], surgical delivery [29], delayed skin-to-skin [30] and attending four or more antenatal care (ANC) contacts [16, 31].

### Breastfeeding and antenatal care

ANC plays a critical role in improving maternal and child health in sub-Saharan Africa as it provides an opportunity to educate and support mothers on maternal and child health issues, including IEBF. In 2016, WHO revised its ANC guidelines, recommending at least eight ANC visits during pregnancy [32, 33]. The policy added three more visits to the third trimester for a total of five third trimester contacts, providing additional opportunities for the integration of nutrition counseling, including EBF promotion, as part of essential maternal healthcare [33]. ANC uptake can also help identify and promptly address high-risk factors, start any necessary treatment and decide mode and place of delivery. During ANC contacts, mothers can receive guidance on the benefits of IEBF and be empowered to identify and address barriers to adopting IEBF [33].

### Study rationale and objective

The extent to which additional ANC contacts has influenced breastfeeding practices in sub-Saharan Africa remains underexplored. The objective of this study is to explore trends in IEBF practices in sub-Saharan Africa since the roll-out of the WHO 2016 ANC policy and investigate associations between number of ANC contacts and IEBF. This study specifically explores differences in 4-7 versus 8 + ANC contacts and breastfeeding practices across sub-Saharan Africa since the roll-out of the 2016 WHO ANC policy. The study aims to provide evidence for policymakers, healthcare providers, and public health organizations to identify what works, refine strategies, improve outcomes, and strengthen the integration of breastfeeding support within ANC services. This study is essential for enhancing the effectiveness of maternal and child health interventions, ultimately contributing to the achievement of global health targets.

## Methods

### Study design

This study used a pooled, cross-sectional design to analyze nationally-representative survey data from the Demographic Health Surveys (DHS) Program for women with a baby aged zero to six months old. Surveys with available data from 2018 to 2023 were included in this study, to reflect a post WHO 2016 policy context.

### Data sources

All data are publicly available and were downloaded from the DHS Program website, http://www.dhsprogram.com. The DHS are standardized national cross-sectional surveys that use a representative multi-cluster sampling design to estimate several measures including maternal and child health related outcomes [34]. A multistage probabilistic sampling methodology is used to select ‘clusters’ and households from geographic-based sampling frames that cover the entire territory of participating countries [35].

### Variables of interest

**Fig. 1:**
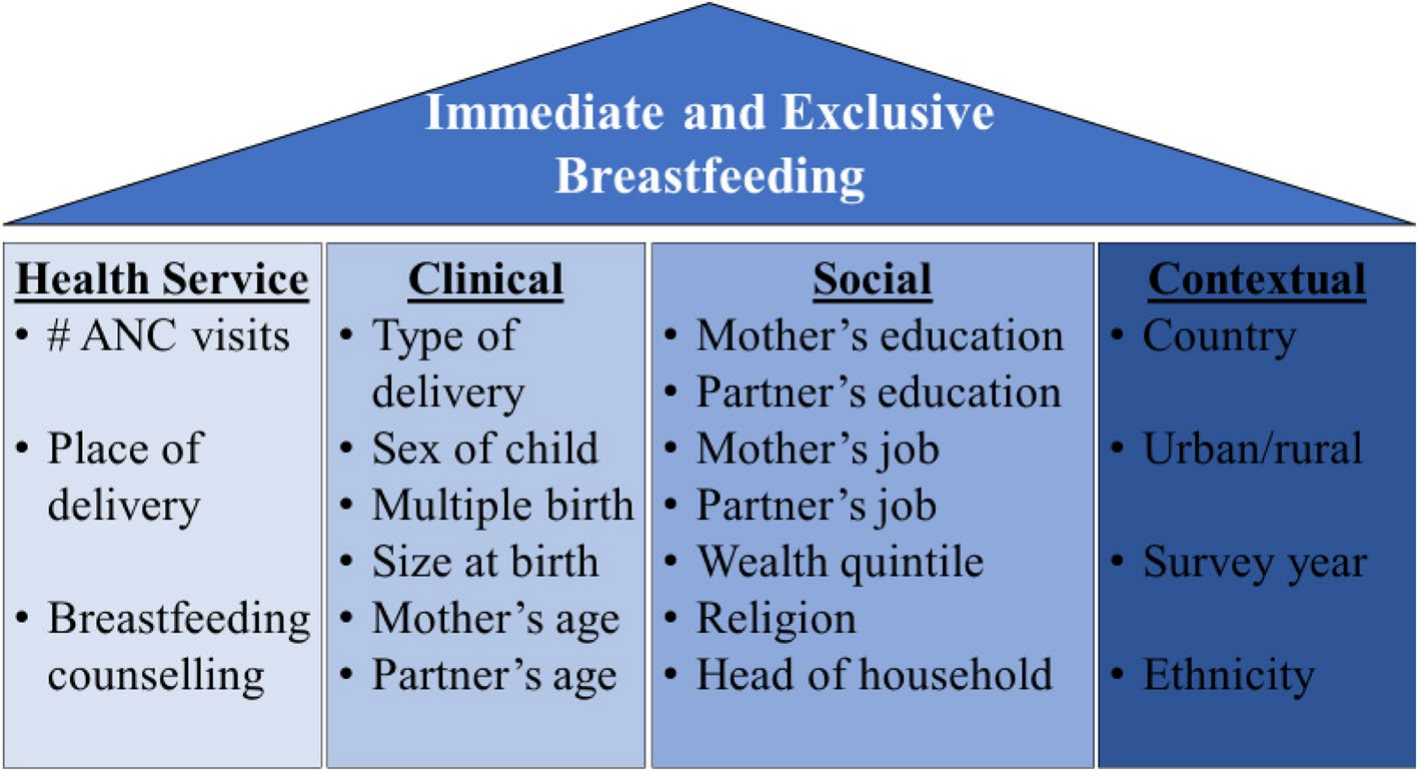
Study variables

Study outcomes included immediate and exclusive breastfeeding for infants aged zero to six months old and were assessed as yes/no. IBF was defined as the infant was breastfed within one hour of birth. EBF was defined as the infant was not fed anything other than breast milk since birth for the first six months. Additional study variables were informed by the UNICEF model of determinants of early initiation of breastfeeding [36] adapted for DHS surveys in Kenya [37] and Nepal [38]. Figure 1 shows the study outcomes of IEBF potentially associated with health service, clinical, social and contextual factors identified from and available in DHS surveys for eligible countries.

The main predictor variable was the number of ANC contacts (0-3, 4-7, and 8+ contacts). Other health service covariates included whether the birth was at home (yes versus no), and breastfeeding counseling within 2 days of delivery (no versus yes). Clinical factors included the type of delivery (vaginal versus cesarean section), sex of child (male versus female), whether the child is a twin (no versus yes) and whether the child was born small (no versus yes), mother and partner’s age group (15-24, 25-34, and 35+ years). Social factors were mother and partner’s education level (primary or less versus above primary) and working status (not working versus working), wealth quintile (first (lowest) to fifth (highest)), religion (Christian versus not), and sex of the head of household (male versus female). Finally, contextual variables included were country, ethnicity, location (urban versus rural), religion (Christian versus Other), and survey year (2018-2023).

### Analysis

Stata version 17 (Stata Corporation, College Station, TX, USA) was used for data management and analysis. The data were weighted using the svyset command to make the data representative of the study population for each country and account for clustering. Country-level chi-square tests were used to explore the bivariate associations between the various covariates and the key outcomes of IBF and EBF. Pooled crude and multivariable logistic regression models were used to explore factors associated with number of ANC contacts and breastfeeding outcomes among all included countries, controlling for the aforementioned covariates. Of note, Ethiopia was missing several variables in the logistic regression analysis, including occupation of partner and mother, education of partner, and birthweight so it was omitted.

## Results

A total of 25,669 individuals across 19 countries were eligible for inclusion (Table 1). Overall, 57.2% of mothers reported IBF, and 42.6% reported EBF while 27.9% reported IEBF (Table 1). Rwanda reported the highest percentage of infants receiving IBF at 86.7%, while Kenya had the highest percentage of EBF at 74.2%. By contrast, Senegal and Gabon had the lowest percentages of IBF (24.4%) and EBF (17.58%) infants, respectively. With the exception of Gambia, Kenya, and Senegal, IBF frequency was higher for all countries than for EBF.

**Table 1:** Description of study participants

	Total	Breastfeeding Status					
		Immediate (*N*=13550)		Exclusive (*N*=11049)		Immediate & Exclusive (*N*=7225)	
	N (%)	(%)	95% CI	(%)	95% CI	(%)	95% CI
TOTAL	25669 (100%)	57.2	56.2-58.2	42.6	41.7-43.5	27.9	27.0-28.6
**Country**							
Burkina Faso	1459 (6%)	61.2	57.5-64.7	45.0	41.4-48.6	29.3	26.0-32.7
Cameroon	1214 (5%)	46.9	42.8-50.8	34.2	30.8-37.7	17.5	15.1-20.2
Côte d'Ivoire	1357 (5%)	44.1	40.0-48.2	29.2	25.2-33.4	10.9	9.03-13.1
Ethiopia	715 (3%)	78.0	72.1-82.8	51.5	45.7-57.1	41.7	35.9-47.7
Gabon	767 (3%)	76.0	69.2-81.5	17.6	13.7-22.2	15.0	11.3-19.6
Gambia	1162 (4%)	33.2	28.9-37.8	46.7	43.0-50.4	16.2	13.7-18.9
Ghana	1153 (4%)	51.1	47.0-55.1	43.5	39.4-47.5	21.9	18.8-25.2
Guinea	1144 (5%)	39.9	35.3-44.5	28.7	25.4-32.0	11.0	8.89-13.6
Kenya	2343 (8%)	66.2	62.3-69.8	74.2	71.8-76.5	64.9	62.1-67.6
Liberia	717 (3%)	66.5	60.5-71.9	45.6	40.0-51.2	30.4	25.9-35.3
Madagascar	1558 (6%)	62.6	59.2-65.7	46.6	43.4-49.8	30.1	27.3-33.0
Mali	1228 (5%)	67.1	63.4-70.4	36.0	32.6-39.4	23.0	20.1-26.1
Mauritania	1463 (6%)	55.5	51.1-59.6	35.1	31.5-38.8	21.3	18.4-24.3
Nigeria	3974 (16%)	43.0	40.7-45.2	24.5	22.8-26.3	11.6	10.4-12.9
Rwanda	907 (4%)	86.7	84.0-88.9	70.8	67.1-74.1	62.1	58.4-65.5
Senegal	757 (3%)	24.4	20.1-29.0	34.4	29.8-39.2	9.2	6.72-12.3
Sierra Leone	1253 (5%)	77.2	73.9-80.1	46.1	42.4-49.6	35.2	31.7-38.8
Tanzania	1290 (5%)	69.4	66.4-72.2	57.2	53.3-61.0	40.8	37.2-44.4
Zambia	1208 (5%)	77.3	74.0-80.1	60.2	56.8-63.3	47.3	43.9-50.6
**Mother characteristics**							
Aged 15-24 years	9677 (37%)	55.8	54.3-57.1	42.6	41.2-43.9	27.2	26.0-28.4
Aged 25-34 years	11395 (45%)	57.4	56.0-58.7	42.6	41.3-43.8	27.8	26.8-29.0
Aged 35+ years	4597 (18%)	59.7	57.7-61.6	42.8	41.0-44.8	29.2	27.5-31.1
Secondary Education	8524 (35%)	57.6	56.1-59.2	44.8	43.3-46.2	29.5	28.1-30.8
Working	14374 (57%)	57.2	56.0-58.4	41.9	40.7-43.0	27.2	26.2-28.3
**Partner Characteristics**							
Aged 15-24 years	1411 (6%)	60.3	57.6-63	46.5	43.2-50.0	32.0	28.9-35.1
Aged 25-34 years	8017 (37%)	56.5	54.9-58.1	44.3	42.8-45.8	29.1	27.7-30.4
Aged 35-4 4 years	7853 (36%)	56.4	55.3-57.6	41.3	39.9-42.7	26.4	25.1-27.6
Aged 45+ years	4433 (20%)	53.6	52.1-55.2	36.7	34.9-38.7	22.1	20.5-23.8
Secondary Education	7577 (31%)	56.2	54.5-57.8	43.8	42.3-45.4	28.7	27.3-30.1
Working	19586 (77%)	55.8	54.7-56.9	41.5	40.5-42.6	26.5	25.6-27.4
**Household Characteristics**							
Poorest	6924 (23%)	56.8	54.8-58.5	43.1	41.5-44.7	28.5	27.0-30.1
Poorer	5711 (22%)	56.6	54.8-58.4	41.6	40.0-43.3	27.5	26.0-29.0
Middle	5157 (20%)	57.5	55.7-59.3	43.4	41.6-45.1	28.6	27.1-30.3
Richer	4430 (19%)	58.3	56.2-60.5	43.6	41.7-45.7	27.9	26.2-29.7
Richest	3447 (16%)	57.2	54.7-59.6	41.2	38.9-43.5	26.3	24.3-28.5
Christian Religion	12294 (48%)	59.8	58.4-61.2	44.6	43.5-45.9	30.2	29.1-31.4
Female Head	20774 (81%)	58.9	57.0-60.9	44.1	42.3-46.0	29.3	27.7-31.0

The majority of mothers and partners represented in the study were 25-34 years of age, working, and had less than a secondary education. Nearly half of households self-identified as Christian (48.4%), and the majority had a male head of household (81.1%). Most households tended to report wealth in the poorest or poorer quintiles (45.1%), as compared to 34.9% in the richer or richest quintiles.

**Fig. 2:**
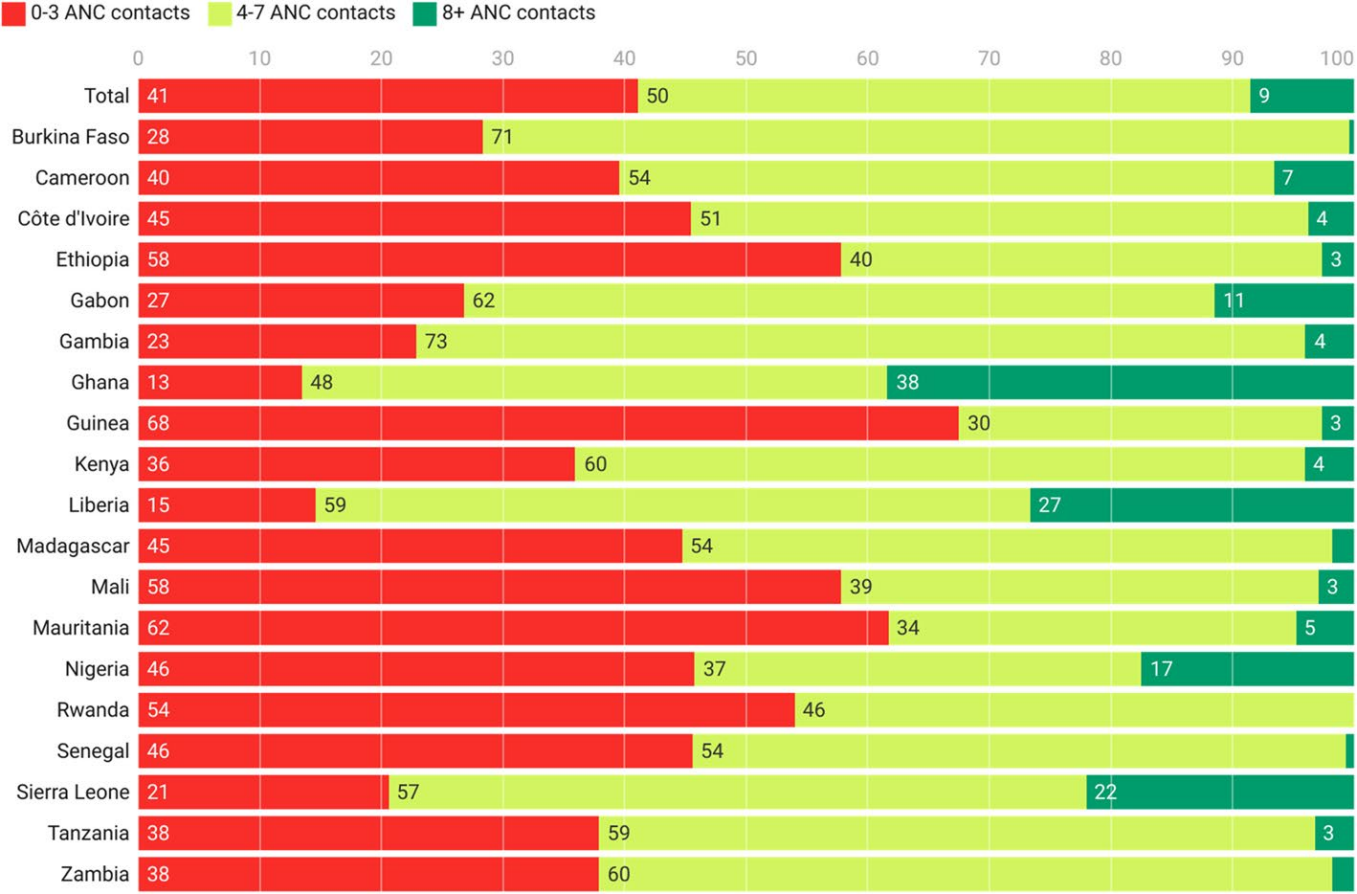
Number of antenatal care contacts across study countries

Across all countries, the majority of participants had 4-7 ANC contacts during their last pregnancy in the previous two years (50.4%) (Fig. 2). Only 8.6% reported 8 or more ANC contacts. However, there was significant variability in the data, ranging from none of the mothers reporting 8 or more ANC contacts in Rwanda, to as high as 38.5% in Ghana.

Table 2 highlights health service, clinical, demographic and contextual factors associated with immediate and exclusive breastfeeding. Factors significantly contributing to increased likelihood of IBF included *health service*: counseling on breastfeeding (OR=1.19); *clinical: *age 25-34 (OR=1.11), age 35 or older (OR=1.13), partner age 45 or older (OR=1.18); *sociodemographic*: wealthiest quintile (OR=1.23), Christian religion (OR=1.17); and *contextual*: Gabon (OR=1.48), Madagascar (OR=1.37), and Rwanda (OR=2.44) compared to Burkina Faso as the reference. Factors that reduced likelihood of IBF included those that were *health service*: homebirth (OR=0.70), *clinical*: Cesarean section (OR=0.23), small infant size at birth (OR=0.79); *sociodemographic*: partner with at least a secondary education (OR=0.89), working partner (OR=0.86); and *contextual*: Cote d’Ivoire (OR=0.57), Gambia (OR=0.18), Mauritania (OR=0.50), and Senegal (OR=0.09) compared to Burkina Faso. Number of ANC contacts, child’s sex, twin status, mother’s education, mother’s working status, and household head sex were not significant.

**Table 2:** Factors associated with immediate and exclusive breastfeeding

	Immediate Breastfed		Exclusively Breastfed	
	aOR	95% CI	aOR	95% CI
**Health Service Factors**				
# ANC contacts (Ref: 4-7)				
0-3	1.01	0.95 1.08	0.92*	0.86 0.98
8+	0.94	0.84 1.05	0.85**	0.76 0.98
Homebirth (Ref: no)	0.70***	0.65 0.76	0.76***	0.70 0.82
Counseled on Breastfeeding (Ref: no)	1.19***	1.11 1.28	1.03	0.96 1.09
**Clinical Factors**				
Cesarean Section (Ref: no)	0.23***	0.20 0.27	0.83**	0.74 0.94
Female child (Ref: male)	1.00	0.94 1.06	0.99	0.94 1.05
Child is a twin (Ref: no)	0.80	0.64 1.00	0.57***	0.46 0.71
Baby born small (Ref: no)	0.79***	0.72 0.85	0.88**	0.81 0.95
Age (Ref: 15-24)				
25-34	1.10*	1.02 1.19	1.11**	1.03 1.20
35+	1.13*	1.02 1.27	1.14*	1.02 1.26
Partner Age (Ref: 15-24)				
25-34	1.09	0.95 1.24	0.99	0.88 1.13
35-44	1.15	0.99 1.33	1.00	0.88 1.15
45+	1.18*	1.00 1.38	0.92	0.79 1.07
**Sociodemographic Factors**				
≥Secondary education (Ref: no)	0.97	0.89 1.05	1.10*	1.01 1.19
Partner education ≥ Secondary (Ref: no)	0.89**	0.82 0.97	1.05	0.97 1.14
Working (Ref: no)	0.96	0.89 1.02	0.93*	0.88 1.00
Partner Working (Ref: no)	0.86**	0.77 0.97	0.99	0.89 1.09
Wealth Quintile (Ref: poorest)				
Poorer	1.05	0.97 1.15	0.91*	0.84 0.99
Middle	1.07	0.97 1.17	0.93	0.85 1.02
Richer	1.05	0.93 1.17	0.89*	0.80 0.99
Richest	1.23**	1.08 1.42	0.76***	0.67 0.87
Christian Religion (Ref: no)	1.17***	1.07 1.28	1.20***	1.10 1.30
Female household head (Ref: male)	1.01	0.93 1.11	0.97	0.89 1.05
**Context Factors**				
Country (Ref: Burkina Faso)				
Cameroon	1.50	0.19 11.80	0.70	0.25 1.98
Côte d'Ivoire	0.57***	0.48 0.67	0.45***	0.37 0.53
Gabon	1.48**	1.11 1.99	0.21***	0.16 0.29
Gambia	0.18***	0.12 0.25	0.89	0.63 1.24
Ghana	1.66	0.20 13.50	1.32	0.44 3.96
Guinea	0.90	0.11 7.05	0.51	0.18 1.44
Kenya	2.78	0.34 22.92	3.34*	1.09 10.21
Liberia	0.71	0.48 1.05	0.77	0.53 1.11
Madagascar	1.37**	1.08 1.73	0.94	0.76 1.16
Mali	3.17	0.40 24.96	0.76	0.27 2.13
Mauritania	0.50***	0.36 0.68	0.52***	0.38 0.70
Nigeria	1.53	0.19 11.96	0.52	0.19 1.47
Rwanda	2.44***	1.65 3.61	1.88***	1.32 2.67
Senegal	0.09***	0.06 0.14	0.35***	0.24 0.52
Sierra Leone	1.23	0.84 1.80	0.72	0.50 1.02
Tanzania	3.43	0.42 28.25	1.56	0.51 4.78
Zambia	4.93	0.63 38.43	1.67	0.60 4.64
Rural location (Ref: urban)	1.01	0.93 1.10	1.03	0.96 1.12
Survey Year (Ref: 2018)				
2019	5.11	0.67 38.84	1.66	0.63 4.39
2020	5.39	0.70 41.32	1.68	0.62 4.49
2021	2.31	0.30 18.05	1.17	0.42 3.25
2022	1.22	0.79 1.89	0.86	0.56 1.30
2023	1.00	N/A	1.00	N/A

Adjusted for the number of ANC contacts, home birth, breastfeeding counseling, cesarean section, sex of child, child is a twin, child was born small, mother and partners’ age group, education level and working status (not working vs. working), wealth quintile, religion, sex of the head of household, country, location, survey year and ethnicity (estimates not shown)

*AOR* Adjusted Odds Ratio, *CI* Confidence Interval, *N/A* Not available, *Ref* Reference

*p<0.05; **p<0.01; ***p<0.001

For EBF, variable effect size direction and significance were generally similar to those for the IBF. Notable exceptions included increased likelihood of EBF for mothers with at least a secondary education (OR = 1.10), and reduced likelihood of EBF for mothers with 0-3 (OR: 0.92) and 8+ (OR: 0.85) ANC visits compared to the reference group of 4-7 visits. In addition, odds of EBF were lower in the poorer (OR = 0.91), richer (OR = 0.89), and richest (OR = 0.76) wealth quintiles compared to the poorest.

Results of the pooled country data indicated that there was no difference in adjusted odds of IBF based on the number of ANC contacts (Table 3). When we looked in each country, we found that for IBF, Gambia had increased odds (OR = 1.91), and Ghana (OR = 0.58) and Nigeria (OR = 0.78) had decreased adjusted odds of IBF with 8 + ANC contacts compared to 4-7. Across all countries, odds of IBF did not significantly differ for 0-3 ANC contacts compared to 4-7 contacts.

**Table 3:** Relationship between the number of antenatal care contacts and immediate and exclusive breastfeeding

	Immediate Breastfeeding AOR 95% CI		Exclusive Breastfeeding AOR 95% CI	
#ANC contacts [ref: 4-7 contacts]	0-3 contacts	8+ contacts	0-3 contacts	8+ contacts
Pooled Total	1.01	0.94	0.92*	0.85**
	0.95 1.08	0.84 1.05	0.86 0.98	0.76 0.94
Burkina Faso	0.99	0.40	1.09	2.25
	0.75 1.29	0.08 1.90	0.84 1.41	0.37 13.60
Cameroon	0.97	1.02	1.61**	0.59
	0.70 1.35	0.55 1.87	1.15 2.24	0.31 1.13
Côte d'Ivoire	0.80	1.19	0.84	0.56
	0.61 1.06	0.56 2.52	0.62 1.13	0.24 1.30
Gabon	1.37	0.88	1.17	0.35
	0.68 2.72	0.28 2.74	0.65 2.12	0.09 1.33
Gambia	0.72	1.91*	0.83	1.01
	0.52 1.01	1.04 3.52	0.60 1.14	0.55 1.84
Ghana	0.76	0.58***	1.12	1.20
	0.49 1.17	0.42 0.79	0.73 1.71	0.89 1.62
Guinea	1.21	1.56	1.01	0.32*
	0.86 1.69	0.64 3.76	0.74 1.40	0.11 0.99
Kenya	1.00	1.01	0.89	1.57
	0.72 1.41	0.40 2.59	0.70 1.14	0.76 3.25
Liberia	1.45	0.91	0.80	0.84
	0.78 2.69	0.55 1.50	0.47 1.35	0.53 1.31
Madagascar	1.13	0.38	1.05	1.68
	0.85 1.50	0.14 1.03	0.80 1.38	0.64 4.41
Mali	1.11	1.13	1.00	0.63
	0.82 1.49	0.48 2.66	0.76 1.32	0.26 1.54
Mauritania	1.04	0.98	0.95	0.94
	0.80 1.35	0.51 1.88	0.74 1.22	0.49 1.80
Nigeria	1.03	0.78*	0.84	0.75*
	0.87 1.22	0.62 0.97	0.70 1.01	0.60 0.95
Rwanda	1.00	1.00	1.17	1.00
	0.61 1.63		0.83 1.64	0.05 1.44
Senegal	1.08	1.00	1.02	1.00
	0.71 1.64	0.63 1.32	0.71 1.46	0.47 1.43
Sierra Leone	1.29	0.92	0.71*	0.61**
	0.82 2.02	0.63 1.32	0.50 0.99	0.45 0.83
Tanzania	1.23	0.88	0.96	0.79
	0.88 1.71	0.36 2.17	0.72 1.28	0.35 1.79
Zambia	0.91	0.98	1.13	1.29
	0.62 1.33	0.24 4.04	0.83 1.52	0.43 3.88

Adjusted for the number of ANC contacts, breastfeeding counseling, home birth, cesarean section, sex of child, child is a twin, child was born small, mother and partners’ age group, education level and working status (not working vs. working), wealth quintile, religion, sex of the head of household, country, location, survey year and ethnicity (estimates not shown)

*AOR* Adjusted Odds Ratio, *CI *Confidence Interval, *Ref *Reference

*p<0.05; **p<0.01; ***p<0.001

However, for EBF, in the pooled country model, women with 0-3 ANC contacts (OR = 0.92) or 8+ (OR = 0.85) had decreased odds compared to women with 4-7 ANC contacts. In country-specific EBF models, Guinea (OR = 0.32), Nigeria (OR = 0.75) and Sierra Leone (OR = 0.61) all had reduced odds of EBF for women with 8 + ANC contacts. Sierra Leone also had reduced odds with 0–3 ANC contacts (OR = 0.71), whereas Cameroon had increased odds (OR = 1.61) of EBF. No other significant associations were found in country-specific models for the number of ANC contacts and EBF.

## Discussion

Drawing on recent DHS cross-sectional survey data from 19 countries across sub-Saharan Africa, we identified wide ranging frequencies of IBF (from 24.4% in Senegal to 86.7% in Rwanda) and EBF (from 17.6% in Gabon to 74.2% in Kenya). This variation is similar to other low- and middle-income countries globally [39], yet most countries remain far below the Global Breastfeeding Collective goal of 70% by 2030 [11]. Notably, in this analysis, the rates of IBF were higher than EBF rates for all countries except Gambia, Kenya, and Senegal. Pooled analysis across all countries showed that having 8 + compared to 4-7 ANC contacts was not significantly associated with IBF or EBF. Study findings demonstrated the intersection of health service (breastfeeding counseling), clinical (cesarean section, and small size at birth), sociodemographic (age, religion and wealth) and contextual factors (country) influencing both IBF and EBF. These findings are consistent with previous research demonstrating the relationship between breastfeeding counseling and IEBF [40] as well as the influence of clinical, sociodemographic, and contextual factors influencing IEBF [41, 42].

### Policy implications

Given that ANC provides a key touchpoint for educating mothers about IEBF [40, 43], policies should prioritize the integration of robust breastfeeding counseling into ANC services. Mandatory breastfeeding counseling policies can ensure that every ANC visit includes dedicated time for breastfeeding education, focusing on both immediate and exclusive breastfeeding, emphasizing its benefits [21], and addressing common misconceptions [18]. Implementation of the timed and targeted counseling model, for instance, by community health workers or other healthcare providers, which has had demonstrated effects on antenatal care as well as breastfeeding outcomes in Uganda, can help connect pregnant women pre, peri-, and postpartum [43, 44]. Policies developing and implementing standardized, evidence-based breastfeeding education curricula for healthcare providers would ensure consistent, high-quality guidance across all health care settings [10]. This could include, for instance, trainings via WHO/UNICEF’s Baby-friendly Hospital Initiative and the Ten Steps to Successful Breastfeeding. In the Democratic Republic of the Congo, a simplified nine-step program was associated with increases in exclusive breastfeeding at six months [45]. Community health worker policies can equip community health workers with knowledge about breastfeeding promotion and support [46], enabling them to reach women in rural or underserved areas [21] who may not have frequent contact with formal healthcare [47, 48]. Study findings corroborated the role of wealth impacting IEBF. As a result, policies may aim to reduce or improve financial agency [49] or eliminate out-of-pocket costs for ANC services, which could increase the number of women attending multiple ANC visits, particularly in low-income regions [32].

### Programmatic implications

The low rates of ANC 8 + and IEBF may be improved by expanding ANC coverage and demand-side initiatives to increase demand for ANC are urgently needed. This could include, for example, implementation of community or group ANC delivery mechanisms [50], which provide pregnant people with social support as well as improve quality and uptake of ANC attendance [51]. Social and behavior change interventions can improve both ANC [32] and IEBF [52]. Large-scale media campaigns aimed at normalizing immediate and exclusive breastfeeding, dispelling myths, and educating families on the importance of breastfeeding for infant health [53]. Given that the study is spread across multiple countries and settings, localized efforts are needed to understand and address the local myths and misconceptions, and campaigns should ensure that messages are tailored to specific audiences and contexts. Campaigns should educate and encourage male partners and other support systems such as mothers and mothers-in-law to be more involved in the breastfeeding process by educating them on how their support is crucial for successful breastfeeding outcomes. Campaigns can also leverage peer education from mothers positioned at IEBF champions in their communities as part of peer counseling groups within communities where trained mothers can provide practical guidance and emotional support to new mothers regarding breastfeeding practices [53]. While the study did not explore norms and perceptions, community engagement and mobilization initiatives to support IEBF may prove beneficial [54].

Evidence that receipt of counseling on breastfeeding was positively associated with IBF but not EBF suggests that additional support beyond the antenatal period may be needed to sustain exclusive breastfeeding. Health system strengthening and provider-level interventions such as digital and mHealth tools and text messaging [55, 56], can improve communication skills and streamline breastfeeding counseling messages during ANC visits to increase prevalence of IBF. Given the relationship between clinical factors and IEBF, frequent high-quality ANC can identify and address clinical conditions that might hinder IEBF. Efforts to improve quality of care during ANC contacts should employ a holistic ecosystem framework [57] to identify specific drivers of provider behavior and clinical skills [58].

### Strengths and limitations

This study had several strengths and limitations. There are limited multi-country studies of the relationship between the number of ANC contacts and breastfeeding in sub-Saharan Africa following the recently updated WHO guidance on ANC visits. Our work directly addresses this gap in the literature by exploring associations with both immediate and exclusive breastfeeding. The study leverages nationally-representative datasets and employs variables from a proven framework. In addition, the study focused on a subset of women with a live birth in the past six months to limit the possibility of recall bias.

Study weaknesses include the cross-sectional nature of the design [35]. Thus, causality cannot be inferred from our data. However, findings from this study lay the groundwork for additional longitudinal designs to examine the potential impact of ANC contacts on subsequent immediate initiation of and exclusive breastfeeding. Additionally, all data is self-reported and may be subject to self-report bias. We also acknowledge that while breastfeeding counselling is an important part of antenatal care, highly motivated mothers may need only one antenatal visit to discuss and adopt IEBF. Finally, there are unexplored variables, such as women’s attitudes and perceptions related to breastfeeding, the content of ANC visits, quality of care, nature of provider-client interactions among others leading to unmeasured confounding. Thus we position our study as one that should inform future longitudinal studies that address the aforementioned gaps.

## Conclusion

We examined trends in breastfeeding practices in sub-Saharan Africa since the rollout of the WHO 2016 ANC policy advocating for 8 + contacts. The study observed low rates of both ANC 8 + and IEBF, with significant variation across countries. No substantial improvements in IEBF were seen between women with 8 + ANC contacts and those with 4-7 ANC contacts. Study findings support relevant policies such as the integration of robust breastfeeding counseling into ANC services and the implementation of standardized, evidence-based breastfeeding education curricula for both facility and community health workers.

## Data Availability

All data are publicly available and were downloaded from the DHS Program website, http://www.dhsprogram.com.

## Abbreviations

ANC: Antenatal Care
DHS: Demographic Health Surveys
EBF: Exclusive Breastfeeding
IBF: Immediate Breastfeeding
IEBF: Immediate and Exclusive Breastfeeding
UNICEF: United Nations Children's Fund
WHO: World Health Organization
